# Effectiveness of BNT162b2 and mRNA-1273 covid-19 vaccines against symptomatic SARS-CoV-2 infection and severe covid-19 outcomes in Ontario, Canada: test negative design study

**DOI:** 10.1136/bmj.n1943

**Published:** 2021-08-20

**Authors:** Hannah Chung, Siyi He, Sharifa Nasreen, Maria E Sundaram, Sarah A Buchan, Sarah E Wilson, Branson Chen, Andrew Calzavara, Deshayne B Fell, Peter C Austin, Kumanan Wilson, Kevin L Schwartz, Kevin A Brown, Jonathan B Gubbay, Nicole E Basta, Salaheddin M Mahmud, Christiaan H Righolt, Lawrence W Svenson, Shannon E MacDonald, Naveed Z Janjua, Mina Tadrous, Jeffrey C Kwong

**Affiliations:** 1 ICES, Toronto, ON, Canada; 2 Dalla Lana School of Public Health, University of Toronto, Toronto, ON, Canada; 3 Public Health Ontario, ON, Canada; 4 Centre for Vaccine Preventable Diseases, University of Toronto, Toronto, ON, Canada; 5 School of Epidemiology and Public Health, University of Ottawa, ON, Canada; 6 Children’s Hospital of Eastern Ontario Research Institute, Ottawa, ON, Canada; 7 Institute of Health Policy, Management, and Evaluation, University of Toronto, Toronto, ON, Canada; 8 Bruyère and Ottawa Hospital Research Institutes, Ottawa, ON, Canada; 9 Department of Medicine, University of Ottawa, Ottawa, ON, Canada; 10 Department of Laboratory Medicine and Pathobiology, University of Toronto, Toronto, ON, Canada; 11 Department of Epidemiology, Biostatistics, and Occupational Health, School of Population and Global Health, McGill University, Montreal, QC, Canada; 12 Vaccine and Drug Evaluation Centre, Department of Community Health Sciences, University of Manitoba, Winnipeg, MB, Canada; 13 Analytics and Performance Reporting Branch, Alberta Health, Edmonton, AB, Canada; 14 Division of Preventive Medicine, Faculty of Medicine and Dentistry, University of Alberta, Edmonton, AB, Canada; 15 School of Public Health, University of Alberta, Edmonton, AB, Canada; 16 Department of Community Health Sciences, Cumming School of Medicine, University of Calgary, Calgary, AB, Canada; 17 Faculty of Nursing, University of Alberta, Edmonton, AB, Canada; 18 British Columbia Centre for Disease Control, Vancouver, BC, Canada; 19 School of Population and Public Health, University of British Columbia, Vancouver, BC, Canada; 20 Women’s College Hospital, Toronto, ON, Canada; 21 Leslie Dan Faculty of Pharmacy, University of Toronto, Toronto, ON, Canada; 22 Department of Family and Community Medicine, University of Toronto, Toronto, ON, Canada; 23 University Health Network, Toronto, ON, Canada

## Abstract

**Objective:**

To estimate the effectiveness of mRNA covid-19 vaccines against symptomatic infection and severe outcomes (hospital admission or death).

**Design:**

Test negative design study.

**Setting:**

Ontario, Canada between 14 December 2020 and 19 April 2021.

**Participants:**

324 033 community dwelling people aged ≥16 years who had symptoms of covid-19 and were tested for SARS-CoV-2.

**Interventions:**

BNT162b2 (Pfizer-BioNTech) or mRNA-1273 (Moderna) vaccine.

**Main outcome measures:**

Laboratory confirmed SARS-CoV-2 by reverse transcription polymerase chain reaction (RT-PCR) and hospital admissions and deaths associated with SARS-CoV-2 infection. Multivariable logistic regression was adjusted for personal and clinical characteristics associated with SARS-CoV-2 and vaccine receipt to estimate vaccine effectiveness against symptomatic infection and severe outcomes.

**Results:**

Of 324 033 people with symptoms, 53 270 (16.4%) were positive for SARS-CoV-2 and 21 272 (6.6%) received at least one dose of vaccine. Among participants who tested positive, 2479 (4.7%) were admitted to hospital or died. Vaccine effectiveness against symptomatic infection observed ≥14 days after one dose was 60% (95% confidence interval 57% to 64%), increasing from 48% (41% to 54%) at 14-20 days after one dose to 71% (63% to 78%) at 35-41 days. Vaccine effectiveness observed ≥7 days after two doses was 91% (89% to 93%). Vaccine effectiveness against hospital admission or death observed ≥14 days after one dose was 70% (60% to 77%), increasing from 62% (44% to 75%) at 14-20 days to 91% (73% to 97%) at ≥35 days, whereas vaccine effectiveness observed ≥7 days after two doses was 98% (88% to 100%). For adults aged ≥70 years, vaccine effectiveness estimates were observed to be lower for intervals shortly after one dose but were comparable to those for younger people for all intervals after 28 days. After two doses, high vaccine effectiveness was observed against variants with the E484K mutation.

**Conclusions:**

Two doses of mRNA covid-19 vaccines were observed to be highly effective against symptomatic infection and severe outcomes. Vaccine effectiveness of one dose was observed to be lower, particularly for older adults shortly after the first dose.

## Introduction

Understanding how efficacy estimates from clinical trials of covid-19 vaccines translate into effectiveness estimates in the real world is crucial, given differences in populations, dosing intervals, and emerging variants.^1^ Because of constraints on the supply of covid-19 vaccines, Canada’s National Advisory Committee on Immunization recommended extending the interval between doses to a maximum of 16 weeks.^2^ With constraints on vaccine supply globally, determining the effectiveness of these vaccines after a single dose versus two doses is important for informing policy for many countries.^1^

We applied the test negative design to linked, population based health databases in Ontario, Canada (population 15 million) to evaluate vaccine effectiveness against symptomatic SARS-CoV-2 infection and severe outcomes (hospital admission or death) associated with SARS-CoV-2 infection for two mRNA vaccines: BNT162b2 (Pfizer-BioNTech) and mRNA-1273 (Moderna).

## Methods

### Study population, setting, and design

We conducted a test negative design study among community dwelling Ontarians who had symptoms consistent with covid-19. The test negative design is comparable to a nested case-control design, with individuals who have symptoms and are tested for the presence of a pathogen of interest serving as the nesting cohort.^1^ ^3^ ^4^ All Ontarians aged ≥16 years, eligible for provincial health insurance, not living in long term care, and who were tested for SARS-CoV-2 between 14 December 2020 and 19 April 2021 were eligible for inclusion. We excluded those who tested positive for SARS-CoV-2 before 14 December 2020 and recipients of the ChAdOx1 (Oxford-AstraZeneca) vaccine. We restricted the analysis to individuals who had at least one relevant covid-19 symptom (based on self-report or observation, such as measured temperature), at the time of testing, which was collected on the SARS-CoV-2 test requisition form (see supplementary methods).

### Data sources and definitions

Using unique encoded identifiers, we linked data from provincial SARS-CoV-2 laboratory testing, covid-19 vaccination, and health administrative datasets and analysed them at ICES (formerly the Institute for Clinical Evaluative Sciences).

### Outcomes

Our first primary outcome was symptomatic SARS-CoV-2 infection, ascertained by real time reverse transcription polymerase chain reaction (RT-PCR) tests on respiratory specimens, including samples from the nasopharynx (most common), nose, throat, saliva, and turbinates.^5^ Using data from the Ontario Laboratories Information System, which captured 91.8% (n=258 207) of all provincially reported cases of laboratory confirmed covid-19 (n=281 261) during the study period, people who tested positive were considered as cases and those who tested negative were considered as controls. Since the dates for symptom onset were inconsistently reported in the Ontario Laboratories Information System, we used the date of specimen collection as the index date. For cases with multiple positive test results, we used the date of the first positive test result. For controls with multiple negative test results, we used the date of a randomly selected negative test result as the index date.

We obtained information on variants and mutations from the Public Health Case and Contact Management system, which contains information on the clinical course of cases and the results of screening tests for N501Y and E484K mutations and whole genome sequencing results that identify specific variant of concern lineages (alpha (B.1.1.7), beta (B.1.351), gamma (P.1)). All RT-PCR positive specimens with cycle threshold values ≤35 were tested for the N501Y mutation (starting 3 February 2021) and the E484K mutation (starting 22 March 2021).^6^ We considered samples with positive N501Y and negative E484K mutations as alpha variants, and samples with positive N501Y and E484K mutations as beta or gamma variants. We combined the latter two lineages for our analysis because there were small numbers of cases identified using whole genome sequencing.

Our second primary outcome was severe disease associated with symptomatic SARS-CoV-2 infection, defined as either hospital admission or death with a recent positive test result, using the earliest of the specimen collection date or the hospital admission or death date as the index date. We identified these outcomes using the Case and Contact Management system (for both hospital admissions and deaths), the Canadian Institute for Health Information’s Discharge Abstract Database (for hospital admissions), and the Ontario Registered Persons Database (for deaths). For hospital admissions identified using the Discharge Abstract Database, a positive test result must have occurred within 14 days before or three days after admission. For deaths identified using the Registered Persons Database, a positive test result must have occurred within 30 days before death or within seven days post mortem. We used the same control group as for the first primary outcome analysis (ie, individuals with symptoms who tested negative for SARS-CoV-2).

### Vaccination against covid-19

BNT162b2 became available in Ontario on 14 December 2020 and mRNA-1273 on 28 December 2020.^7^ The initial vaccination phase prioritised high risk populations such as older adults living in communal settings, healthcare workers (including non-patient facing staff working in healthcare institutions), adults living in Indigenous communities, and adults aged ≥80 years.^7^ Ontario had initially followed the manufacturers’ recommended dosing schedules (ie, a 21 day interval for BNT162b2 and a 28 day interval for mRNA-1273), but because of disruptions to vaccine supply in late January 2021, the interval was extended to 35-42 days for everyone except older adults living in communal settings, and Indigenous people. In early March, Ontario adopted the National Advisory Committee on Immunization’s recommendation to delay administering the second dose by up to 16 weeks for most individuals.^8^ ^9^ Eligibility expanded over time, taking into account both age (ie, graduated expansion by decreasing age) and other high risk populations, such as people with certain health conditions and their care givers, certain essential frontline workers, and those aged ≥18 years living or working in communities with a high incidence of covid-19 (ie, those disproportionately affected by covid-19 and where transmission was still high). Adherence to these eligibility criteria varied across regions. As of 19 April 2021, 28% of adults in Ontario had received at least one dose of a covid-19 vaccine.^10^ Comprehensive documentation of all covid-19 vaccination events in Ontario, including product, date of being administered, and dose number, is recorded in real time into COVaxON, a centralised covid-19 vaccine information system. We used the COVaxON file containing events up to 25 April 2021 for these analyses, which likely had records of all vaccinations delivered by 19 April 2021.

### Covariates

We obtained age, sex, and postal code of residence as of 14 December 2020 from the Registered Persons Database. We obtained the number of RT-PCR tests for each participant during the three months before 14 December 2020 from the Ontario Laboratories Information System to use as a proxy for highly tested individuals at increased risk of exposure to SARS-CoV-2 infection (eg, healthcare workers and care givers of long term care residents, who must also undergo serial SARS-CoV-2 testing). To capture temporal changes in viral activity and regional vaccine roll-out, we grouped dates of testing into two week periods. We determined the presence of comorbidities that increase the risk of severe covid-19,^11^ identified from various databases using validated algorithms and commonly accepted diagnostic codes, which have been described elsewhere.^12^ Receipt of influenza vaccination (a proxy for health behaviours) was ascertained during the 2019/20 or 2020/21 influenza season, or both, using physician and pharmacist billing claims in the Ontario Health Insurance Plan and Ontario Drug Benefit databases, respectively. We determined the public health unit of residence using the postal code and Statistics Canada Postal Code Conversion File Plus (version 7B) and grouped the units into larger regions. From 2016 census data we obtained information at the ecological level of dissemination area on four important social determinants of health (median household income, proportion of the working population employed as non-health essential workers (those unable to work from home), average number of people in each dwelling, and proportion of the population who self-identified as a visible minority).^13^ Dissemination areas generally contain 400-700 people. Supplementary eTable 1 provides details of these covariates.

### Statistical analysis

To compare characteristics between test positive cases and test negative controls and between vaccinated and unvaccinated individuals, we conducted descriptive analyses and calculated standardised differences.

We used multivariable logistic regression models to estimate the odds ratio, comparing the odds of vaccination between test positive cases and test negative controls (with unvaccinated people as reference group). We estimated unadjusted and adjusted odds ratios accounting for all listed covariates. These covariates were selected a priori based on their known associations with SARS-CoV-2 infection or severity and receipt of a covid-19 vaccine^2^ ^11^ ^14^ and were assessed as potential confounders (supplementary eTable 2).^15^ Vaccine effectiveness was calculated using the formula: vaccine effectiveness=(1−odds ratio)×100%. We assumed those without information on exposures, outcomes, or covariates in ICES’ data holdings to not have the exposure, outcome, or covariate, and they were categorised as such within the analyses.

For the primary analysis, we estimated overall vaccine effectiveness (for both mRNA vaccines combined) for those who received only one dose by their index date and those who received two doses by their index date. We considered index dates within varying intervals after vaccination.

Vaccine effectiveness was also estimated ≥14 days after the first dose (among those who only received one dose) and ≥7 days after the second dose,^16^ stratified by vaccine product (BNT162b2 or mRNA-1273), age group (16-39, 40-69, and ≥70 years), sex, presence of any comorbidity, epidemic wave (index dates 14 December 2020 to 7 February 2021, representing wave 2 in Ontario; 8 February 2021 to 21 March 2021, representing the period between wave 2 and wave 3; and 22 March 2021 to 19 April 2021, representing wave 3), and variant (earlier variant versus alpha versus beta or gamma). We also estimated vaccine effectiveness by varying intervals after vaccination, stratified by age group.

We repeated these analyses for severe outcomes, with adjustments to the intervals after vaccination due to reduced sample sizes. For example, we evaluated vaccine effectiveness for the entire period (≥0 days) after receipt of the second dose.

Finally, to assess whether systematic differences between vaccinated and unvaccinated individuals were adequately controlled for in the main analyses, in a sensitivity analysis we assessed vaccine effectiveness against symptomatic infection and severe outcomes by varying intervals among only those who were vaccinated (treating people vaccinated 0-13 days before the test as reference group).^17^

All analyses were conducted using SAS version 9.4 (SAS Institute, Cary, NC). Tests were two sided, with P*<*0.05 considered as significant.

### Patient and public involvement

Although study participants contributed in important ways to this research, it was not feasible to involve them in the design, conduct, reporting, or dissemination plans of our research. We did not involve members of the public in this research owing to resource and time constraints.

## Results

**Fig 1:**
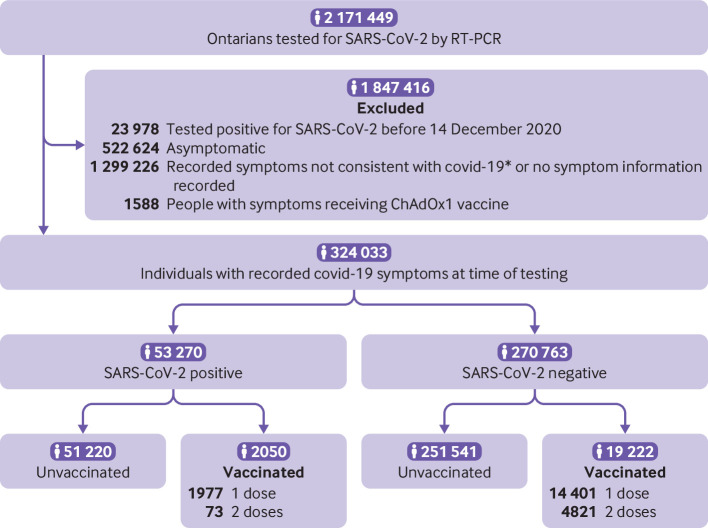
Community dwelling people aged ≥16 years included in tested cohort between 14 December 2020 and 19 April 2021 in Ontario, Canada. *Recorded symptoms not consistent with covid-19 (eg, anxiety, cancer, falls). RT-PCR=reverse transcription polymerase chain reaction; ChAdOx1=Oxford-AstraZeneca vaccine

Overall, 2 171 449 unique individuals were tested for SARS-CoV-2 from 14 December 2020 to 19 April 2021. After excluding those who had SARS-CoV-2 infection before the study period and those who had received the ChAdOx1 vaccine, 60.5% of those remaining did not have symptoms consistent with covid-19 or had no information on symptoms recorded in the Ontario Laboratories Information System, 24.4% were recorded as asymptomatic, and 15.1% had symptoms consistent with covid-19 recorded at the time of testing (fig 1). Grouped together, those with covid-19-like symptoms and those deemed asymptomatic had similar characteristics to the remaining individuals, except for covid-19 vaccine uptake, public health unit region, and number of previous SARS-CoV-2 tests (supplementary eTable 3).

Of the 324 033 people with symptoms who were tested, 53 270 (16.4%) tested positive for SARS-CoV-2, 42 567 (79.9%) had information available on tests for variants, 21 272 (6.6%) had received at least one dose of mRNA vaccine, and 4894 (1.5%) had received two doses (table 1). Among test positive cases, 2479 (4.7%) had a severe outcome, of whom 2035 were admitted to hospital and 444 died. Most cases admitted to hospital tested positive before or on the admission date (1728, 84.9%) and nearly all tested positive before death. Test positive cases were more likely to be male, to reside in Peel region or Toronto, and to have had no SARS-CoV-2 tests during the three months before the vaccination programme, less likely to have received an influenza vaccine, and more likely to reside in neighbourhoods with lower income, more people in each dwelling, and greater proportions of essential workers and people from visible minorities (table 1). Vaccinated people were older, less likely to be male, and more likely to have had multiple SARS-CoV-2 tests during the three months before the vaccination programme, a comorbidity, and received an influenza vaccine. Compared with recipients of the mRNA-1273 vaccine, recipients of the BNT162b2 vaccine were younger, more likely to be female, and less likely to have a comorbidity (supplementary eTable 4). The distribution of these vaccine products also differed by public health unit regions. Most participants (77% for BNT162b2, 76% for mRNA-1273) had received only one dose by the index date. Supplementary eFigure 1 presents the distribution of vaccine product over the study period.

**Table 1:** Characteristics of participants with symptoms tested for SARS-CoV-2 between 14 December 2020 and 19 April 2021 in Ontario, Canada. Values are numbers (percentages) unless stated otherwise

Characteristics	SARS-CoV-2 status		Standardised difference*	Vaccination status		Standardised difference*
	Positive (n=53 270)	Negative (n=270 763)		≥1 dose mRNA vaccine (n=21 272)	Unvaccinated (n=302 761)	
Vaccine dose:						
At least 1	2050 (3.8)	19 222 (7.1)	-	-	-	-
2	73 (0.1)	4821 (1.8)	-	-	-	-
Positivity to SARS-CoV-2 type:	-	-	-	2050 (9.6)	51 220 (16.9)	-
Earlier variant	-	-	-	579 (2.7)	27 510 (9.1)	-
Alpha (B.1.1.7)	-	-	-	807 (3.8)	12 282 (4.1)	-
Beta (B.1.351) or gamma (P.1) (variants with E484K mutation)	-	-	-	98 (0.5)	1291 (0.4)	-
Mean (SD) age (years)	42.4 (17.1)	43.2 (17.8)	0.04	51.8 (20.8)	42.4 (17.3)	0.49
Age group (years):						
16-29	15 175 (28.5)	72 238 (26.7)	0.04	3457 (16.3)	83 956 (27.7)	0.28
30-39	10 024 (18.8)	59 326 (21.9)	0.08	4011 (18.9)	65 339 (21.6)	0.07
40-49	9642 (18.1)	46 225 (17.1)	0.03	3287 (15.5)	52 580 (17.4)	0.05
50-59	9460 (17.8)	40 874 (15.1)	0.07	3112 (14.6)	47 222 (15.6)	0.03
60-69	5279 (9.9)	27 342 (10.1)	0.01	2261 (10.6)	30 360 (10.0)	0.02
70-79	2426 (4.6)	14 888 (5.5)	0.04	2272 (10.7)	15 042 (5.0)	0.21
≥80	1264 (2.4)	9870 (3.6)	0.07	2872 (13.5)	8262 (2.7)	0.40
Males	25 993 (48.8)	112 501 (41.5)	0.15	6013 (28.3)	132 481 (43.8)	0.33
Public health unit region†:						
Central East	2624 (4.9)	29 194 (10.8)	0.22	1969 (9.3)	29 849 (9.9)	0.02
Central West	8322 (15.6)	48 419 (17.9)	0.06	3853 (18.1)	52 888 (17.5)	0.02
Durham	1433 (2.7)	8583 (3.2)	0.03	522 (2.5)	9494 (3.1)	0.04
Eastern	689 (1.3)	15 147 (5.6)	0.24	1087 (5.1)	14 749 (4.9)	0.01
North	1753 (3.3)	31 321 (11.6)	0.32	2251 (10.6)	30 823 (10.2)	0.01
Ottawa	417 (0.8)	3144 (1.2)	0.04	446 (2.1)	3115 (1.0)	0.09
Peel	13 515 (25.4)	32 981 (12.2)	0.34	2395 (11.3)	44 101 (14.6)	0.10
South West	7562 (14.2)	39 316 (14.5)	0.01	3885 (18.3)	42 993 (14.2)	0.11
Toronto	12 458 (23.4)	45 540 (16.8)	0.16	3462 (16.3)	54 536 (18.0)	0.05
York	4278 (8.0)	15 995 (5.9)	0.08	1323 (6.2)	18 950 (6.3)	0.00
Biweekly period of test:						
14 Dec 2020 to 27 Dec 2020	4139 (7.8)	27 456 (10.1)	0.08	13 (0.1)	31 582 (10.4)	0.48
28 Dec 2020 to 10 Jan 2021	6870 (12.9)	26 993 (10.0)	0.09	335 (1.6)	33 528 (11.1)	0.40
11 Jan 2021 to 24 Jan 2021	4864 (9.1)	26 747 (9.9)	0.03	1068 (5.0)	30 543 (10.1)	0.19
25 Jan 2021 to 7 Feb 2021	3539 (6.6)	24 276 (9.0)	0.09	1204 (5.7)	26 611 (8.8)	0.12
8 Feb 2021 to 21 Feb 2021	3595 (6.7)	24 800 (9.2)	0.09	1031 (4.8)	27 364 (9.0)	0.17
22 Feb 2021 to 7 Mar 2021	3539 (6.6)	30 760 (11.4)	0.17	1491 (7.0)	32 808 (10.8)	0.13
8 Mar 2021 to 21 Mar 2021	5134 (9.6)	32 776 (12.1)	0.08	2790 (13.1)	35 120 (11.6)	0.05
22 Mar 2021 to 4 Apr 2021	8338 (15.7)	35 910 (13.3)	0.07	4814 (22.6)	39 434 (13.0)	0.25
5 Apr 2021 to 19 Apr 2021	13 252 (24.9)	41 045 (15.2)	0.24	8526 (40.1)	45 771 (15.1)	0.58
No of tests in past three months:						
0	43 713 (82.1)	189 786 (70.1)	0.28	11 588 (54.5)	221 911 (73.3)	0.40
1	7151 (13.4)	54 827 (20.2)	0.18	4338 (20.4)	57 640 (19.0)	0.03
≥2	2406 (4.5)	26 150 (9.7)	0.20	5346 (25.1)	23 210 (7.7)	0.49
Any comorbidity‡	23 212 (43.6)	127 974 (47.3)	0.07	12 218 (57.4)	138 968 (45.9)	0.23
2019-2020 and/or 2020-21 influenza vaccine	13 751 (25.8)	89 395 (33.0)	0.16	9587 (45.1)	93 559 (30.9)	0.30
Household income fifth†§:						
1 (lowest)	11 878 (22.3)	47 944 (17.7)	0.11	3750 (17.6)	56 072 (18.5)	0.02
2	11 154 (20.9)	51 470 (19.0)	0.05	4146 (19.5)	58 478 (19.3)	0.00
3	11 477 (21.5)	52 628 (19.4)	0.05	4233 (19.9)	59 872 (19.8)	0.00
4	10 146 (19.0)	56 676 (20.9)	0.05	4513 (21.2)	62 309 (20.6)	0.02
5 (highest)	8359 (15.7)	60 774 (22.4)	0.17	4540 (21.3)	64 593 (21.3)	0.00
Essential workers fifth (%)†¶:						
1 (0-32.5)	6440 (12.1)	50 664 (18.7)	0.18	3917 (18.4)	53 187 (17.6)	0.02
2 (32.5-42.3)	11 225 (21.1)	60 040 (22.2)	0.03	4664 (21.9)	66 601 (22.0)	0.00
3 (42.3–49.8)	11 106 (20.8)	56 108 (20.7)	0.00	4468 (21.0)	62 746 (20.7)	0.01
4 (50.0-57.5)	11 576 (21.7)	52 849 (19.5)	0.05	4211 (19.8)	60 214 (19.9)	0.00
5 (57.5-100)	12 519 (23.5)	49 067 (18.1)	0.13	3859 (18.1)	57 727 (19.1)	0.02
No (range) of people per dwelling fifth†:						
1 (0–2.1)	5781 (10.9)	51 852 (19.2)	0.23	4277 (20.1)	53 356 (17.6)	0.06
2 (2.2–2.4)	6641 (12.5)	52 326 (19.3)	0.19	4219 (19.8)	54 748 (18.1)	0.04
3 (2.5–2.6)	5633 (10.6)	37 229 (13.7)	0.10	3020 (14.2)	39 842 (13.2)	0.03
4 (2.7–3.0)	12 967 (24.3)	63 774 (23.6)	0.02	4874 (22.9)	71 867 (23.7)	0.02
5 (3.1–5.7)	21 833 (41.0)	63 459 (23.4)	0.38	4709 (22.1)	80 583 (26.6)	0.10
Self-identified visible minority fifth (%)† **:						
1 (0.0-2.2)	4437 (8.3)	51 919 (19.2)	0.32	4133 (19.4)	52 223 (17.2)	0.06
2 (2.2-7.5)	5752 (10.8)	55 124 (20.4)	0.27	4592 (21.6)	56 284 (18.6)	0.07
3 (7.5-18.7)	7223 (13.6)	51 122 (18.9)	0.14	3982 (18.7)	54 363 (18.0)	0.02
4 (18.7-43.5)	10 718 (20.1)	53 691 (19.8)	0.01	3974 (18.7)	60 435 (20.0)	0.03
5 (43.5-100)	24 736 (46.4)	56 876 (21.0)	0.56	4438 (20.9)	77 174 (25.5)	0.11

* Values >0.10 are considered clinically relevant.

† Sum of counts does not equal column total because of individuals with missing information (<1.0%) for this characteristic.

‡ Comorbidities include chronic respiratory diseases, chronic heart diseases, hypertension, diabetes, immunocompromising conditions due to underlying diseases or treatment, autoimmune diseases, chronic kidney disease, advanced liver disease, dementia/frailty, and history of stroke or transient ischaemic attack.

§ Includes variable cut-off values in each city/census area to account for cost of living. A dissemination area (DA) being in the first fifth means it is among the lowest 20% of DAs in its city by income.

¶ Percentage of people in area working in sales and service occupations; trades, transport and equipment operators, and related occupations; natural resources, agriculture, and related production occupations; and occupations in manufacturing and utilities. Census counts for people are randomly rounded up or down to the nearest number divisible by 5, which results in some minor imprecision.

** Percentage of people in area who self-identified as a visible minority. Census counts for people are randomly rounded up or down to the nearest number divisible by 5, which results in some minor imprecision.

**Fig 2:**
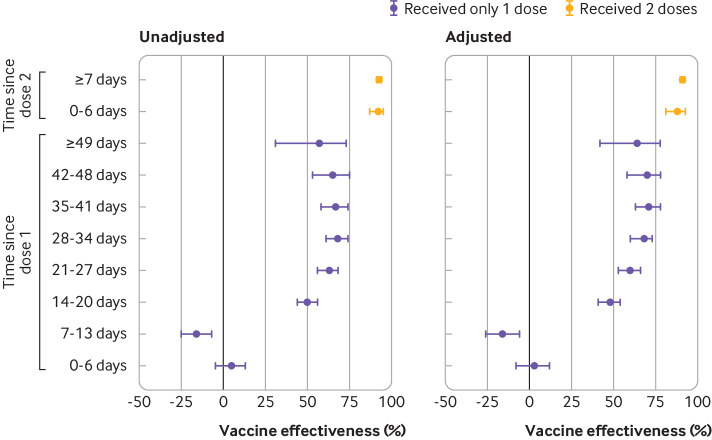
Unadjusted and adjusted vaccine effectiveness estimates of covid-19 mRNA vaccines (BNT162b2, mRNA-1273) against laboratory confirmed symptomatic SARS-CoV-2 infection by various intervals, between 14 December 2020 and 19 April 2021 in Ontario, Canada. Models were adjusted for age, sex, public health unit region, biweekly period of test, number of SARS-CoV-2 tests in the three months before 14 December 2020, presence of any comorbidity increasing the risk of severe covid-19, receipt of influenza vaccination in current or previous influenza season, and fifths of neighbourhood level household income, number of people in each dwelling, proportion of people employed as non-health essential workers, and self-identified visible minority

**Fig 3:**
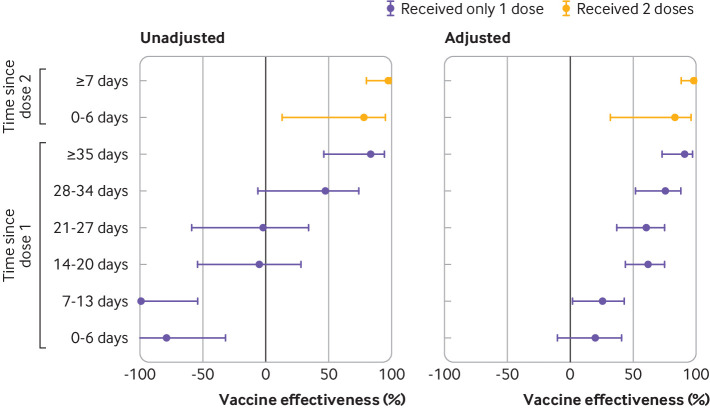
Unadjusted and adjusted vaccine effectiveness estimates of covid-19 mRNA vaccines (BNT162b2, mRNA-1273) against severe outcomes (hospital admission or death) associated with laboratory confirmed symptomatic SARS-CoV-2 infection by various intervals, between 14 December 2020 and 19 April 2021 in Ontario, Canada. Models were adjusted for age, sex, public health unit region, biweekly period of test, number of SARS-CoV-2 tests in the three months before 14 December 2020, presence of any comorbidity increasing the risk of severe covid-19, receipt of influenza vaccination in current or previous influenza season, and fifths of neighbourhood level household income, number of people in each dwelling, proportion of people employed as non-health essential workers, and self-identified visible minority

Against symptomatic infection, adjusted vaccine effectiveness observed ≥14 days after only one dose was 60% (95% confidence interval 57% to 64%). This increased from 48% (41% to 54%) at 14-20 days to a plateau of 71% (63% to 78%) at 35-41 days (fig 2, supplementary eTable 5). A 16% increase in risk of symptomatic infection was observed 7-13 days after one dose (vaccine effectiveness −16%, −26% to −6%), but no increase was observed 0-6 days after one dose. Vaccine effectiveness observed ≥7 days after two doses was 91% (89% to 93%). Against severe outcomes of hospital admission or death, vaccine effectiveness observed ≥14 days after one dose was 70% (60% to 77%), increasing from 62% (44% to 75%) at 14-20 days to 91% (73% to 97%) at ≥35 days, whereas vaccine effectiveness observed ≥7 days after two doses was 98% (88% to 100%) (fig 3, supplementary eTable 5).

**Fig 4:**
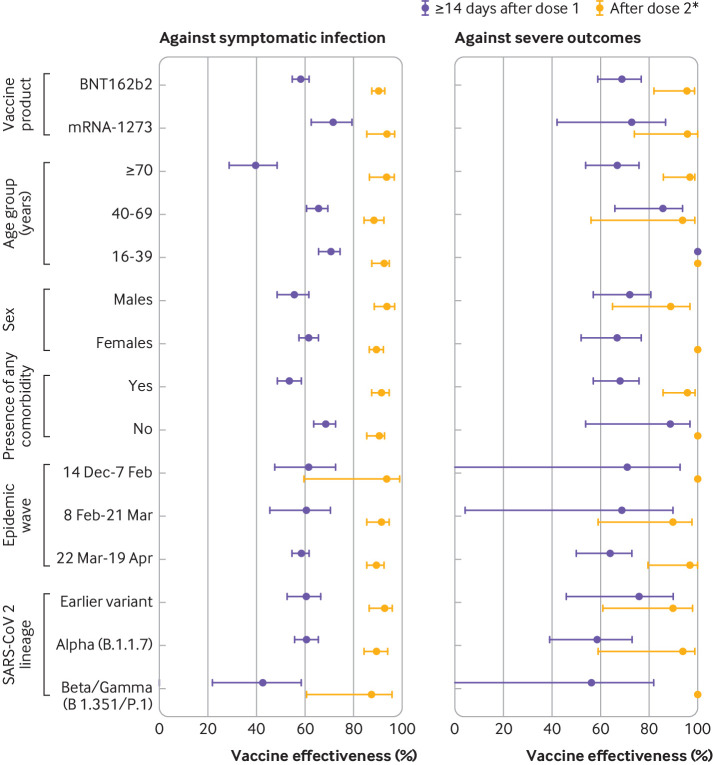
Adjusted vaccine effectiveness estimates ≥14 days after dose 1 (for those who received only one dose) and ≥0 days after dose 2 of covid-19 mRNA vaccines (BNT162b2, mRNA-1273) by various factors, including vaccine product, patient characteristics, epidemic wave, and SARS-CoV-2 lineage against laboratory confirmed symptomatic SARS-CoV-2 infection and severe outcomes (hospital admission or death) between 14 December 2020 and 19 April 2021 in Ontario, Canada. Models were adjusted for age, sex, public health unit region, biweekly period of test, number of SARS-CoV-2 tests in the three months before 14 December 2020, presence of any comorbidity increasing the risk of severe covid-19, receipt of influenza vaccination in current or previous influenza season, and fifths of neighbourhood level household income, number of people living in each dwelling, proportion of people employed as non-health essential workers, and self-identified visible minority (unless adjusted variable was used for stratification). *For vaccine effectiveness estimates against symptomatic SARS-CoV-2 infection, this interval was ≥7 days after dose 2. Against severe outcomes, vaccine effectiveness was evaluated for the entire period (≥0 days) after receipt of the second dose owing to the small number of outcomes. For subgroup analyses by characteristic and SARS-CoV-2 lineage, individuals vaccinated with either mRNA vaccine were included

In subgroup analyses of vaccine effectiveness against symptomatic infection, higher effectiveness was observed ≥14 days after only one dose of mRNA-1273 versus one dose of BNT162b2 (which was consistent across all age groups), for younger people versus adults aged ≥70 years, for those with no comorbidities versus those with comorbidities, and against the earlier variant and alpha variant versus beta or gamma variant (although 95% confidence intervals for vaccine effectiveness estimates for variants overlapped) (fig 4, supplementary eTable 6). Vaccine effectiveness estimates observed ≥7 days after two doses were, however, high (all ≥88%) and comparable across all subgroups, including against variants with the E484K mutation. Against severe outcomes, higher vaccine effectiveness was observed ≥14 days after one dose for those aged 16-39 years, but effectiveness estimates after two doses were mostly similar across subgroups (fig 4, supplementary eTable 7).

**Fig 5:**
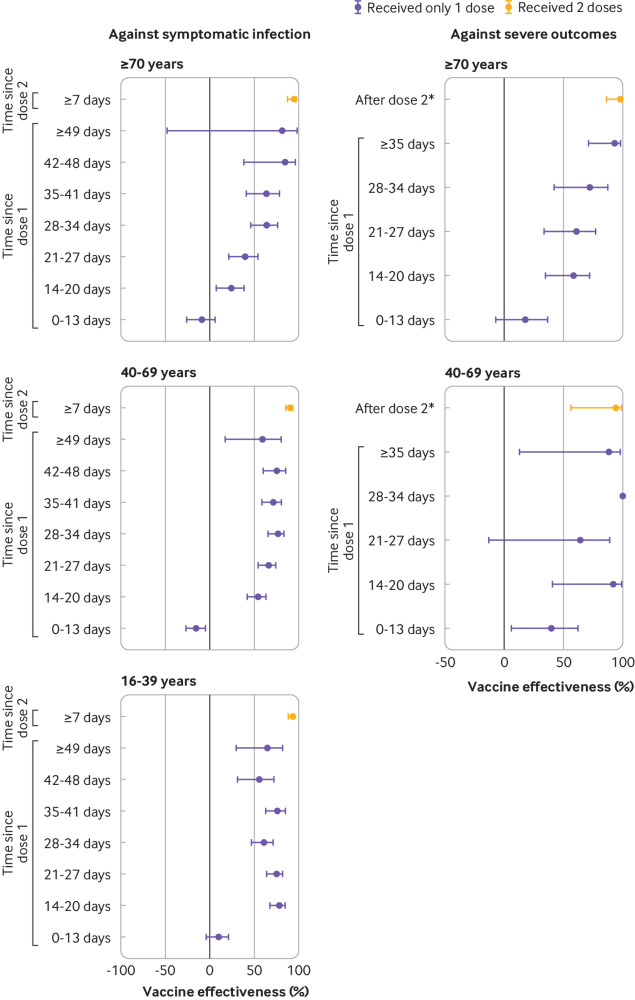
Adjusted vaccine effectiveness estimates ≥14 days after dose 1 and ≥0 days after dose 2 of covid-19 mRNA vaccines (BNT162b2, mRNA-1273) against laboratory confirmed symptomatic SARS-CoV-2 infection and severe outcomes (hospital admission or death) by age group, between 14 December 2020 and 19 April 2021 in Ontario, Canada. Models were adjusted for age, sex, public health unit region, biweekly period of test, number of SARS-CoV-2 tests in the three months before 14 December 2020, presence of any comorbidity increasing the risk of severe covid-19, receipt of influenza vaccination in current or previous influenza season, and fifths of neighbourhood level household income, number of people living in each dwelling, proportion of people employed as non-health essential workers, and self-identified visible minority (unless adjusted variable was used for stratification). *For vaccine effectiveness estimates against symptomatic SARS-CoV-2 infection, this interval was ≥7 days after dose 2. Against severe outcomes, vaccine effectiveness was evaluated for the entire period (≥0 days) after receipt of the second dose owing to the small number of outcomes

Among adults aged ≥70 years, vaccine effectiveness against symptomatic infection after one dose was observed to be 64% (46% to 76%) at 28-34 days and 85% (38% to 97%) at 42-48 days, whereas comparable effectiveness estimates were achieved sooner after one dose for younger people (fig 5, supplementary eTable 8). Furthermore, vaccine effectiveness against severe outcomes was observed to be similar at ≥35 days after one dose (93%, 71% to 98%) as after two doses (97%, 86% to 99%).

In the sensitivity analysis restricted to vaccinated people with those vaccinated 0-13 days before the index date serving as the reference group, vaccine effectiveness estimates against symptomatic infection were observed to be similar to those of the main analyses (supplementary eTable 5). For hospital admissions and deaths, however, the estimates differed for earlier vaccination intervals.

## Discussion

Using the test negative study design, which mitigates selection bias from differences in health seeking behaviour between vaccinated and unvaccinated people, we estimated high (>90%) vaccine effectiveness of mRNA vaccines BNT162b2 and mRNA-1273 against symptomatic SARS-CoV-2 infection with full vaccination (≥7 days after a second dose), and moderate (about 50-70%) vaccine effectiveness with partial vaccination (≥14 days after only one dose). Estimates for both full and partial vaccination were about 10 percentage points higher against hospital admission or death than against symptomatic infection. Vaccine effectiveness generally was observed to increase over time after one dose; however, we also observed a slightly increased risk of symptomatic infection on days 7-13 after one dose, compared with no vaccination. In subgroup analyses, we observed lower vaccine effectiveness against symptomatic infection in adults aged ≥70 years and those with comorbidities, but a higher effectiveness after one dose of mRNA-1273 than after one dose of BNT162b2. Vaccine effectiveness was, however, observed to be consistently high across subgroups for fully vaccinated people, and also for older adults after longer intervals from a first dose.

### Comparison with other studies

Our findings for fully vaccinated people are comparable with efficacy estimates from clinical trials and other real world effectiveness estimates reported in a range of settings.^16^ ^18^ ^19^ ^20^ ^21^ ^22^ ^23^ ^24^ ^25^ ^26^ ^27^ ^28^ ^29^ ^30^ ^31^ Existing evidence for effectiveness estimates of one dose of mRNA vaccines from observational studies is heterogeneous,^16^ ^27^ ^30^ ^31^ ^32^ with estimates for symptomatic infection ranging from 57% (95% confidence interval 50% to 63%)^16^ to 72% (58% to 86%)^31^ and post hoc calculations from efficacy trials about 90%.^33^ ^34^ Similar heterogeneity has been found among one dose effectiveness estimates in older adults,^25^ ^32^ ^35^ with estimates generally lower for older adults after one dose,^16^ ^32^ and increasing over time. In our analysis we observed an effectiveness against symptomatic infection of 63% (95% confidence interval 40% to 72%) ≥49 days after only one dose, in keeping with the findings of several other studies.^16^ ^30^ In addition, we found that one dose of mRNA-1273 was associated with significantly higher effectiveness against symptomatic infection than one dose of BNT162b2. Differences in characteristics between recipients of the two vaccines might explain this finding, but similar results were also found in another Canadian province.^36^ Findings were, however, inconsistent in other studies that compared vaccine effectiveness between products after one dose; some found a trend towards higher effectiveness against infection using mRNA-1273,^37^ ^38^ whereas others found no difference.^39^ ^40^ However, the populations in each of these studies were more homogeneous than ours (eg, adults aged ≤40 years, healthcare workers, veterans). Our analysis also reflects extant evidence that vaccine effectiveness against symptomatic infection^16^ and covid-19 associated hospital admissions^19^ ^23^ increases to high levels after a second dose, even in older adults. Lastly, our finding that two doses of mRNA vaccines was not associated with appreciable vaccine escape by lineage alpha or variants with the E484K mutation (beta and gamma) is notable.

In our study, we observed an increased risk of infection 7-13 days after vaccination. Other studies also found an increased risk of SARS-CoV-2 infection up to 14 days after one dose.^25^ ^31^ ^41^ ^42^ This could be due to an increase in exposures to SARS-CoV-2 after vaccination. Individuals might assume that they are protected against infection immediately after vaccination and engage in higher risk behaviours before a sufficient immune response has developed. Indeed, about 20% of the US public believe that protection is conferred either immediately or 1-2 weeks after the first vaccine dose.^43^ Future studies should examine the potential role of behavioural changes after the first dose of covid-19 vaccines. This finding could also be due a higher baseline risk of infection among those who were initially prioritised to receive the vaccine, which might not have been adequately controlled for in our models. A vaccine effectiveness estimate of close to 0% at 0-6 days after one dose, however, provides a level of validation that we had accounted for the differences between vaccinated and unvaccinated people.

### Limitations of this study

This study has some limitations. Firstly, our study sample was limited to those with symptoms of covid-19 recorded in the Ontario Laboratories Information System, which decreased our potential sample size considerably, from 2 171 449 people who were tested for SARS-CoV-2 to 324 033 who had relevant covid-19 symptoms recorded in the information repository. Not all laboratories in Ontario currently have the information technology infrastructure to submit information on symptoms (or documentation of asymptomatic testing) recorded on the SARS-CoV-2 laboratory requisition form into the Ontario Laboratories Information System. Thus, the generalisability of our findings to the broader population is uncertain and we could not estimate vaccine effectiveness against asymptomatic infection. However, the per cent positivity of our study sample (53 270/324 033=16.4%) did not differ much from that observed in Ontario during the study period (281 261/2 171 449=13.0%), and we would expect positivity to be higher for people with symptoms than those without symptoms. We also acknowledge that those with no information on symptoms recorded in the Ontario Laboratories Information System might have had symptoms at the time of testing, and those recorded as asymptomatic might have subsequently developed symptoms. In addition, covid-19 vaccination status might also be collected on the laboratory requisition form. This could bias the true vaccine effectiveness estimate, depending on whether symptoms were more likely to be documented on requisition forms for vaccinated people who ultimately test positive for SARS-CoV-2 (this would bias vaccine effectiveness towards the null) or less likely to be recorded (this would bias vaccine effectiveness away from the null). To minimise this selection bias, traditional test negative design studies collect vaccination status among all people with symptoms consistent with the pathogen under study. However, the congruence of our findings for fully vaccinated people with extant studies provides some reassurance that any underestimation or overestimation of vaccine effectiveness is likely to be small. Secondly, because the date of symptom onset is largely unavailable in the Ontario Laboratories Information System, and the Case and Contact Management system only has information on people who test positive, we used specimen collection date as the index date. This might have led to some vaccinated people being classified into an incorrect dose-to-index interval because symptom onset would have occurred several days before being tested. The impact of earlier intervals on vaccine effectiveness estimates if using the specimen collection date depends on the test results for vaccinated people (eg, vaccine effectiveness for earlier intervals would be overestimated if there were more vaccinated test positive cases and underestimated if there were more vaccinated test negative controls) or whether the lag between symptom onset and specimen collection dates resulted in misclassification of vaccinated people (false negatives), which would overestimate vaccine effectiveness for earlier intervals. Furthermore, we could not limit the study population to those tested within 10 days of symptom onset, a commonly used inclusion criterion for test negative studies. Prolonging the interval between symptom onset and testing increases the likelihood of false negative cases, which lowers vaccine effectiveness estimates. However, 89% of cases with dates for both symptom onset and specimen collection documented in the Case and Contact Management system (not the source of symptom data for this study) were tested within 10 days of symptom onset. Thirdly, our results might have been affected by outcome misclassification of severe outcomes due to unlinked case records and incomplete capture of severe outcomes in the Case and Contact Management system, and delays in identifying hospital admissions in the Discharge Abstract Database (which depends on people being discharged) and deaths in the Registered Persons Database. The direction of bias to vaccine effectiveness estimates depends on whether data completeness and lags are differential between vaccinated and unvaccinated test positive cases. For example, if vaccination status ascertained during the case management process influenced the degree of data collection (eg, if more complete among vaccinated cases), vaccine effectiveness would be biased towards the null, or if unvaccinated cases have a prolonged hospital admission because of more severe course of illness, their hospital record would not be available for analysis and vaccine effectiveness would be biased towards the null. Fourthly, some of our covariates might be subject to measurement error. We used frequency of previous SARS-CoV-2 tests as a proxy to identify those at higher risk of exposure (and increased likelihood to be targeted for early vaccination). However, we did not include point of care tests because they are incompletely captured in the Ontario Laboratories Information System. Furthermore, since access to testing is variable, we might not have adequately controlled for this situation. Finally, we might not have adequately accounted for confounding bias with the covariates that were available in the study databases, especially against hospital admissions and deaths.

### Conclusions

Our findings suggest that older people and those with comorbidities might benefit from risk based recommendations to minimise delays before a second dose of mRNA covid-19 vaccine. However, increasing protection against severe outcomes—arguably the more important outcomes—with increasing time after a first dose provides support for delaying the second dose in settings with constraints on vaccine supply. Mathematical modelling could be done to show how, particularly for jurisdictions with limited vaccine supply, vaccines should be distributed to maximise the protection of populations (eg, the relative benefits of providing second doses earlier to older populations versus providing more first doses to younger populations who respond better to one dose and thereby leading to more rapid achievement of herd immunity by maximising coverage with one dose). Since vaccine effectiveness against symptomatic infection after one dose is only moderate, and among older adults appears to be modest even at 14-20 days, people need to be informed that besides the absence of benefit during the first two weeks (and likely longer for older adults) after one dose of a mRNA covid-19 vaccine, they should continue to adhere to recommended public health measures, such as wearing a mask, physical distancing, and avoidance of social gatherings.

**What is already known on this topic**

Clinical trials and real world effectiveness studies have shown that mRNA covid-19 vaccines are protective against symptomatic SARS-CoV-2 infections and associated severe outcomes such as hospital admission and death
Greater protection is conferred after two doses administered at least three weeks apart

**What this study adds**

Protection against symptomatic infection and severe outcomes increases gradually after one dose of an mRNA covid-19 vaccine
Although lower vaccine effectiveness was observed in older adults shortly after one dose, levels of protection similar to those of younger people were observed after longer intervals
These findings support delaying second doses when vaccine supply is limited

## Data Availability

The study dataset is held securely in coded form at ICES. While legal data sharing agreements between ICES and data providers (eg, healthcare organisations and government) prohibit ICES from making the dataset publicly available, access might be granted to those who meet prespecified criteria for confidential access, available at www.ices.on.ca/DAS (email das@ices.on.ca). The full dataset creation plan and underlying analytic code are available from the authors upon request, understanding that the computer programs might rely upon coding templates or macros that are unique to ICES and are therefore either inaccessible or require modification.

## Web extra

Extra material supplied by authors

Supplementary information: additional methods, eTables 1-8, and eFigures 1 and 2

## Notes

Contributors: HC and JCK designed and oversaw the study. SH and HC obtained the data and conducted all analyses (dataset and variable creation and statistical modelling). BC contributed to data analyses and data preparation for the symptomatic dataset. SN, MES, HC, and JCK drafted the manuscript. All authors contributed to the analysis plan, interpreted the results, critically reviewed and edited the manuscript, approved the final version, and agreed to be accountable for all aspects of the work. JCK is the guarantor. The corresponding author attests that all listed authors meet authorship criteria and that no others meeting the criteria have been omitted.

The corresponding author (the manuscript’s guarantor) affirms that the manuscript is an honest, accurate, and transparent account of the study being reported; that no important aspects of the study have been omitted; and that any discrepancies from the study as planned have been explained.

Dissemination to participants and related patient and public communities: As the personal identifying information of participants have been removed from the study dataset, it is not possible to send the results of this study to participants. However, the results from this manuscript have been made publicly available through a preprint server and will be further disseminated by ICES through social media channels and news media.

Provenance and peer review: Not commissioned; externally peer reviewed.
